# Retraction Note: LAMP2 expression dictates azacytidine response and prognosis in MDS/AML

**DOI:** 10.1038/s41375-020-0969-8

**Published:** 2020-07-14

**Authors:** Alix Dubois, Nathan Furstoss, Anne Calleja, Marwa Zerhouni, Thomas Cluzeau, Coline Savy, Sandrine Marchetti, Mohamed Amine Hamouda, Sonia Boulakirba, François Orange, Sandra Lacas-Gervais, Jean-Michel Karsenti, Nicolas Mounier, Jérôme Tamburini, Alexandre Puissant, Frederic Luciano, Arnaud Jacquel, Patrick Auberger, Guillaume Robert

**Affiliations:** 1 Université Côte d’Azur, Nice, France; 2 Inserm U1065, C3M, Team: Myeloid Malignancies and Multiple Myeloma, Nice, France; 3 Equipe Labellisée par la Fondation ARC, Paris, France; 4 CHU de Nice, Département d’Hématologie Clinique, Nice, France; 5 Centre Commun de Microscopie Appliquée, Nice, France; 6 Institut Cochin, CNRS, UMR 8104, INSERM U1016, Université Paris Descartes, Paris, France; 7 Inserm U944, Institut Universitaire d’Hématologie, Hôpital St Louis, Paris, France

**Keywords:** Myelodysplastic syndrome, Chemotherapy

Retraction to: *Leukemia*

10.1038/s41375-018-0336-1

The authors have decided to retract this article [1] because after publication they discovered that the SKM1-R, OCI-AML2 and MOLM13 cell lines used were cross-contaminated with murine hematopoietic cells. The results obtained using these cell lines are therefore unreliable. The data presented on primary patient samples remains valid.

All authors agree with this retraction.
